# Predicting the Binding Patterns of Hub Proteins: A Study Using Yeast Protein Interaction Networks

**DOI:** 10.1371/journal.pone.0056833

**Published:** 2013-02-19

**Authors:** Carson M. Andorf, Vasant Honavar, Taner Z. Sen

**Affiliations:** 1 Department of Computer Science, Iowa State University, Ames, Iowa, United States of America; 2 Bioinformatics and Computational Biology Program, Iowa State University, Ames, Iowa, United States of America; 3 United States Department of Agriculture-Agriculture Research Service Corn Insects and Crop Genetics Research Unit, Ames, Iowa, United States of America; 4 Department of Genetics, Development and Cell Biology, Iowa State University, Ames, Iowa, United States of America; University of Cincinnati College of Medicine, United States of America

## Abstract

**Background:**

Protein-protein interactions are critical to elucidating the role played by individual proteins in important biological pathways. Of particular interest are hub proteins that can interact with large numbers of partners and often play essential roles in cellular control. Depending on the number of binding sites, protein hubs can be classified at a structural level as singlish-interface hubs (SIH) with one or two binding sites, or multiple-interface hubs (MIH) with three or more binding sites. In terms of kinetics, hub proteins can be classified as date hubs (i.e., interact with different partners at different times or locations) or party hubs (i.e., simultaneously interact with multiple partners).

**Methodology:**

Our approach works in 3 phases: Phase I classifies if a protein is likely to bind with another protein. Phase II determines if a protein-binding (PB) protein is a hub. Phase III classifies PB proteins as singlish-interface versus multiple-interface hubs and date versus party hubs. At each stage, we use sequence-based predictors trained using several standard machine learning techniques.

**Conclusions:**

Our method is able to predict whether a protein is a protein-binding protein with an accuracy of 94% and a correlation coefficient of 0.87; identify hubs from non-hubs with 100% accuracy for 30% of the data; distinguish date hubs/party hubs with 69% accuracy and area under ROC curve of 0.68; and SIH/MIH with 89% accuracy and area under ROC curve of 0.84. Because our method is based on sequence information alone, it can be used even in settings where reliable protein-protein interaction data or structures of protein-protein complexes are unavailable to obtain useful insights into the functional and evolutionary characteristics of proteins and their interactions.

**Availability:**

We provide a web server for our three-phase approach: http://hybsvm.gdcb.iastate.edu.

## Introduction

Proteins are the principal catalytic agents, structural elements, signal transmitters, transporters and molecular machines in cells. Functional annotation of proteins remains one of the most challenging problems in functional genomics, however, our evolving understanding of a proteins' interaction partners helps in functional annotation of proteins [1]. Protein-protein interactions are therefore critical to elucidating the role played by individual proteins in important biological pathways. Such networks are typically constructed using high throughput techniques (e.g., yeast two-hybrid (Y2H) experiments).

Our current understanding of protein-protein interaction networks is quite limited for a variety of reasons. The challenge of reliable and complete determination of the interactome is far from being fully addressed due to the high rate of false positives. These false positives are associated with high throughput experiments, the low coverage of solved co-crystal structures in the Protein Data Bank (PDB), and the difficulty of obtaining reliable negative evidence that a protein does not interact with one or more other proteins. For example, Y2H experiments focus on pair-wise interactions between proteins and provide, at best, rather indirect evidence for higher order interactions e.g., those that require three proteins to come together to form a complex. Even in the case of pairwise interactions, Y2H experiments only provide evidence that a pair of proteins is likely to interact *in vitro*, without offering any insights into the physical basis of such interactions, or whether such interactions may actually occur *in vivo* [2]–[6]. It is well known that data from high-throughput Y2H experiments are notoriously noisy and suffer from a high false positive rate [7]. The high-quality datasets tend to have low-coverage e.g., it is estimated up to 95% of the human interactome is unmapped [8]. Furthermore, whether a particular protein-protein interaction is experimentally observed depends on the specific experimental conditions. It is therefore critical to validate the putative interactions between proteins suggested by Y2H experiments using additional experimental or computational studies. As a result, considerable amount of recent work has focused on creating high-quality interaction datasets by systematically removing errors and low-quality interactions or by combining multiple sources of evidence [2], [3], [8], [9]. Hence, there is considerable interest in reliable prediction of protein-protein interactions.

Protein-protein interaction networks are usually represented and visualized as graphs in which the nodes correspond to the proteins and edges denote their possible pairwise interactions. Such a representation is simply not rich enough to encode interactions that involve more than two proteins, nor do they distinguish between them. Furthermore, a single target protein can interact with a large number of partners: some of these interactions may be mutually exclusive because of competition between potential binding partners for the same interaction sites on the target protein. Other interactions may be simultaneously possible, and in many instances, even mutually cooperative, i.e., binding of one partner to the target protein may prepare the target for binding to a second partner [10], [11]. Distinguishing between these various types of interactions is essential for uncovering the physical basis of interactions of a protein with other proteins, engineering the protein surfaces to manipulate synthetic pathways, or for designing drugs that bind specific targets [12]–[14]. However, answering such questions is extremely difficult in the absence of direct experimental evidence, e.g., structures of complexes formed by a protein when it interacts with one or more other proteins or results of site-specific mutation experiments that identify the protein surface residues that play essential roles in such interactions. Unfortunately, experimental determination of protein-protein complexes or of binding sites is notoriously time-consuming and expensive. Hence, there is a growing interest in computational tools that provide useful insights into various structural aspects of protein interactions from protein sequence alone.

**Figure 1:**
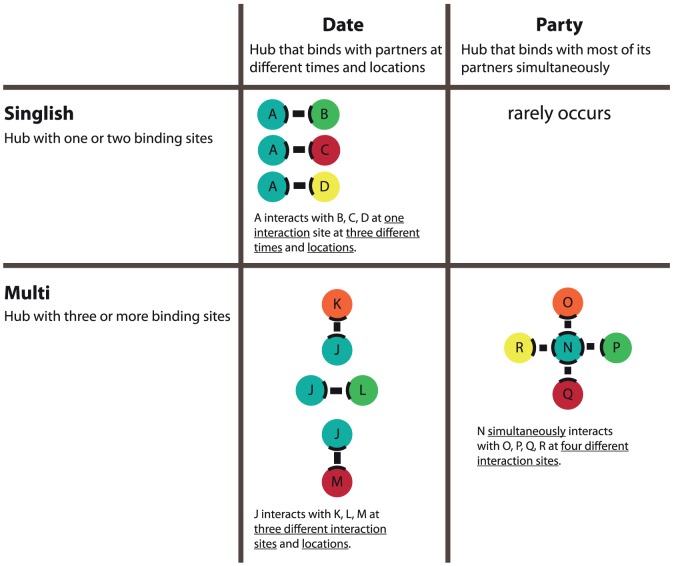
Descriptions of the singlish-date, singlish-party, multi-date, and multi-party classes. Descriptions for each type of hub are described below. The rows of the table represent the singlish and multi-interface hub proteins. The columns represent the date and party hubs. The intersection of the column and row displays a picture showing examples of the type and number of interfaces involved for that class.

Of particular interest in this study are hub proteins, i.e., proteins that interact with large numbers of partners [15]. It is worth noting that “large numbers of partners” is a relative term and is arbitrarily defined. In several studies, hub proteins are defined as those with 5 or more interaction partners [15]–[18]. The choice of five (as opposed to some other number) or more interacting partners as the defining characteristic of hub proteins is somewhat arbitrary. The quality of protein-protein interaction data (false positives, incomplete coverage) presents additional challenges in categorizing proteins into hubs and non-hubs. These difficulties notwithstanding, hub proteins have been reported to play essential roles in cellular control and tend to be highly conserved across species [19]. Mutations in hub proteins can potentially disrupt its interactions with its many interaction partners, which can turn out to be lethal for the cell's survival [20]–[22]. Hence, it is especially important to understand physical and structural basis of interactions of hub proteins with their partners. Recent studies suggest that hubs are more diverse than previously thought and show striking differences in number of binding sites and kinetics of binding. Kim et al. [16] combined three-dimensional structure information, known domain-domain interaction data, and protein-interaction data to define two types of hub protein structures. The first type of hub proteins, called singlish interface hubs (SIH), interacts with multiple partners at one or two binding sites. Because the interactions rely on binding events at one or two binding sites, interactions with the different partners tend to be mutually exclusive. The second type of hub proteins, called multiple-interface hubs (MIH), interacts with multiple interaction partners through more than two binding sites (See Figure 1). Recent studies [16], [22]–[30] have explored the roles of SIH and MIH in protein-protein interactions and hence protein function. Kim et al. [16], who were among the first to analyze the properties of SIH and MIH proteins, found that MIH were twice as likely (compared to SIH) to be *essential* for survival and perhaps as a consequence, more conserved across species with implications for determining the evolutionary rates for protein hubs. They also found that MIH proteins are more likely to be members of large stable structural complexes. SIH and MIH also differ in terms of network expansion during evolution: SIH appear to follow the canonical preferential gene duplication model whereas MIH do not [16]. A recent study showed SIH tend to display higher degrees of disorder than MIH [28]. Table 1 summarizes the results of previous studies [16], [28] that have compared the properties of SIH and MIH.

**Table 1:** Properties of singlish and multiple-interface yeast protein hubs.

Properties	Singlish-interface	Multiple-interface
Essential	No	Yes
Conserved	No	Yes
Co-expression	Limited	High
3D Structure	Smaller, less stable	Larger, stable
Canonical preferential gene duplication	Yes	No
Disorder	High	Low

The properties for each type of interface are listed based on observed tendencies seen in Dataset 3 [16], [28].

Hub proteins can also be classified based on the kinetic mode of interaction. Han et al. [31] recently described an expression-based classification model for hub proteins. This classification is based on a bimodal distribution of co-expression of hub proteins with their interaction partners [31]. Date hubs tend to display expression levels that have low correlation with those of their interaction partners (and tend to bind different partners at different time points or locations). Conversely, party hubs tend to exhibit expression levels that have high degree of correlation with those of their interaction partners (and tend to interact simultaneously with the partners). See Figure 1 for an illustration of date hubs versus party hubs. The analysis of party and date hubs provides additional insights into the structure of the underlying proteome and interactome. For example, date hubs contribute to global network stability and connectivity by acting as a inter-module linkers [10] that serve as regulators, mediators, or adapters. In contrast, party hubs act as intra-module linkers that coordinate a specific process or assist the formation of a specific protein complex [31], [32]. In these intermolecular interactions, the residues that contribute the most to binding (hot spots) for date hubs tend to be spatially near each other (forming hot regions) [25]. Date hubs are likely to evolve faster than party hubs [33]. Table 2 summarizes the conclusions of previous studies that have compared the properties of date hubs and party hubs [25], [31], [33], [34]. The differences between the two types of hub proteins strongly suggest that they might play different functional roles. SIH tend to be date hubs whereas MIH tend to be party hubs [16]; but there are exceptions. It should be no surprise that SIH tend be date hubs: the number of binding sites that a hub protein has limits the number of partners with which it can interact at the same time. However, the converse does not necessarily hold, i.e., not every date hub is a SIH. A date hub may only have one or two concurrent interactions at any given time, but each of these interactions may involve different binding sites. Hence a date hub can in general be a SIH or a MIH. Similarly a party hub tends to be a MIH, since many concurrent interactions require many interaction sites, but a MIH can be a party hub or a date hub based on the interaction kinetics. Recent studies have focused on the role of hubs in interaction networks and in particular, the differences in SIH versus MIH and date hubs versus party hubs [22]–[24], [26], [27], [29]–[31], [35], [36].

**Table 2:** Properties of date and party yeast protein hubs.

Properties	Date	Party
Evolutionary rate	Faster	Slower
Interactome connectivity	Intermodule	Intramodule
Structural interaction	Few interaction sites	Many interaction sites
Hot spots	More organized in hot regions	Less organized in hot regions
Hot regions	Covers a larger fraction of the interface region, larger number of distinct hot regions	Covers a smaller fraction of the interface region, smaller number of distinct hot regions

The properties for each type of interface are listed based on observed tendencies seen in Dataset 4 [25], [31], [33].

Experimental characterization of hub proteins in terms of their structural and kinetic characteristics requires knowledge of the structures of complexes formed by such proteins in interaction with their binding partners [35], [37]. Because of the prohibitive cost and effort needed to determine the structures of complexes formed by hub proteins with their binding partners and the interfaces that mediate such interactions, there is an urgent need for reliable methods for predicting the structural and kinetic characteristics of hubs from sequence information alone. In particular, there is a growing interest in purely sequence-based computational methods for discriminating between simultaneously possible versus mutually exclusive interactions[27], [31], [38], [39] and predicting the number of binding sites available on the surface of a protein.

There has been considerable work on machine learning approaches for distinguishing hub proteins from non-hub proteins [40]–[42]. Mirzarezaee et al. have recently proposed methods for distinguishing between date hubs (that interact with one partner at a time) and party hubs (that simultaneously interact with many partners) [15] using 17 features including 4 composition measurements, grouping of 48 physicochemical properties, six GO term features, domain occurrence, disordered regions, and position specific scoring matrices (PSSM). They reported correlation coefficients of 0.74 for both date and party hubs. In light of these results, a natural question to ask is whether similar or better performance can be achieved from information based solely on the sequence of the hub protein.

**Figure 2:**
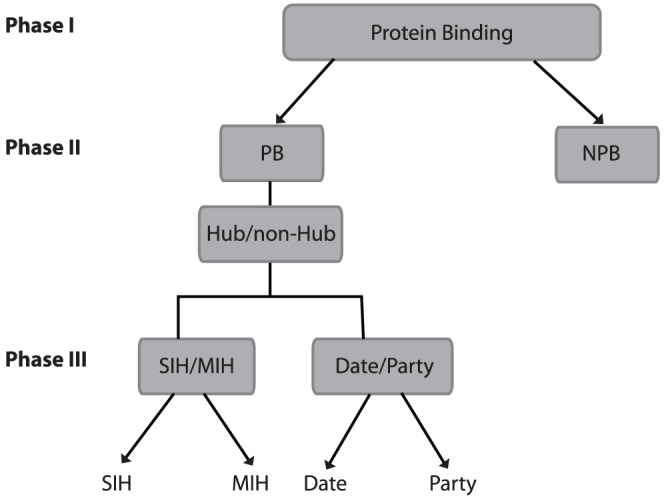
Three-phase method to predict protein-binding proteins, hub proteins, singlish interface/multiple interface (SIH/MIH), and Date/Party hubs. Phase I predicts if a protein physically binds with other proteins (protein-binding (PB) versus non-protein-binding (NPB)). If a protein is predicted to be a PB protein in Phase I, that protein is further classified in Phase II and Phase III. Phase II uses sequence similarity to determine the potential number of interaction sites for the input sequence and if that protein is likely to be a hub protein. Phase III applies methods for predicting both structural (singlish vs. multiple) and kinetic (date vs. party) classifications of protein hub proteins. All methods for each of the three phases make predictions from sequence alone.

**Figure 3:**
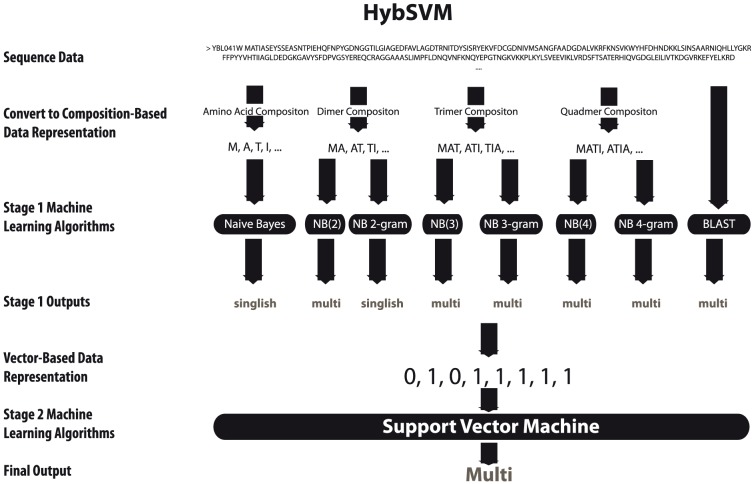
HybSVM method. *HybSVM* is a two-stage machine learning method. The first step of the algorithm is to convert sequence data into a composition-based data representation (monomer, dimer, trimer, and tetramer). These four new data representations are used as inputs to 7 machine learning algorithms based on the NB(k) and NB k-gram approaches (Stage 1). An eighth method based on PSI-BLAST is applied to the original sequence data. The outputs of each of the eight outputs are converted into a binary vector of length 8. The resulting vector is used as input to a SVM to produce the final output (Stage 2).

Against this background, we introduce a three-phase machine learning approach (See Figure 2). Phase I predicts if a protein physically binds with other proteins (protein-binding (PB) versus non-protein-binding (NPB)). If a protein is predicted to be a PB protein, that protein goes through the second and third phase of predictions. Phase II uses sequence similarity to determine the potential number of interaction sites for the input sequence based on a weighted-average of the number of interactors of the top scoring BLAST hits. Phase III applies methods for predicting both structure (singlish vs. multiple) and kinetics (date vs. party) classifications of protein-binding proteins using information derived from only the sequence of the protein (See Figure 3). Our experiments show that our method is able to predict whether a protein is a protein-binding protein with an accuracy of 94%, 0.93 area under a ROC curve (AUC) and a correlation coefficient of 0.87; identify hubs from non-hubs with 100% accuracy for 30% of the data (with the rest being flagged as putative hubs or putative non-hubs depending on the sequence similarity to known hubs/non-hubs in our dataset); distinguish date hubs/party hubs with 69% accuracy and AUC of 0.68; and SIH/MIH with 89% accuracy, 0.85 AUC. The method can be used even in settings where reliable protein-protein interaction data, or structures of protein-protein complexes are unavailable, to obtain useful insights into the functional and evolutionary characteristics of proteins and their interactions. In addition, our method does not rely on computationally expensive multiple sequence alignments, the presence of functional or structural domains, or additional functional annotations (e.g. GO terms), allowing for fast and updateable predictions.

It should be noted that categorizing hub proteins into structural and kinetic classes presents many challenges. SIH and date proteins are defined by the absence of concurrent interaction partners or interaction sites. However, it is difficult to reliably determine the absence of interaction between a protein and one or more putative interaction partners because of the lack of experimental data under a broad range of conditions. It is thus possible that some proteins labelled as SIH in our dataset are in fact MIH where not all interaction partners have been identified. Conversely, because of the high false positive rates associated with high-throughput experiments, some proteins labelled as MIH or party hubs are in fact SIH. These sources for errors in the protein-protein interaction data need to be kept in mind in interpreting the results of our study as well as other similar analyses of protein-interaction data.

A web server for the three-phase approach for automated PB/NPB, SIH/MIH, and date/party prediction is available at http://hybsvm.gdcb.iastate.edu.

## Results and Discussion

Our approach to classifying proteins based on binding patterns is a 3-phase approach: Phase I predicts if a protein is likely to bind with another protein, i.e., protein-binding (PB). Phase II determines if a protein-binding protein is a hub. Phase III classifies PB proteins as singlish-interface versus multiple-interface hubs and date versus party hubs, based on sequence information alone. We present results of experiments for each of the three phases.

In this study, we use a simple encoding of protein sequences using the probability distribution short (*k*-letter) subsequences (*k*-grams) of amino acids. In our experiments, we used values of k ranging from *k* = 1 (amino acid composition) through k = 4 (dimers, trimers, and tetramers). Larger values of *k* were not considered, because we run out of data to reliably estimate the model parameters. We use a range of standard machine learning methods implemented in Weka version 3.6.0: J4.8 version [43] of the C4.5 decision tree learning algorithm (Decision Tree) [44], SMO version [45] of the support vector machine (SVM) [46] with a polynomial kernel, Multilayer Perception neural network (ANN) [43], and Naïve Bayes algorithm [43]. In addition, in Phase I and III, we use a two-stage ensemble classifier, *HybSVM*, which uses an SVM to combine the outputs of a set of predictors. We compare the results of predictors trained using machine learning methods with two baseline methods: the first baseline method classifies proteins based on the number of SCOP [47], [48] and PFAM [49] domains (domain-based method) present in the sequence. The second baseline method classifies each protein based on the class-label of its nearest PSI-BLAST hit. To evaluate predictors constructed using machine learning we used 10-fold cross-validation. Because any single measure e.g., accuracy, provides at best partial information about the performance of a predictor, we use a set of measures including accuracy, precision, recall, correlation coefficient, F-measure, and area under the Receiver Operating Characteristic (ROC) curve. Additional details can be found in the Methods section of the paper.

### Predicting protein binding proteins (Phase I)

To evaluate our method to discriminate proteins that bind to other proteins from those that bind to other substrates (e.g., small ligands), we assembled Dataset 1, which consists of 5,010 proteins including 3,418 proteins that bind to one or more proteins and 1,592 that bind to small ligands, but are not known to bind to other proteins. As mentioned in the introduction, creating a set of proteins that do not bind to any other protein is a difficult challenge due to low-coverage and high false-positive rates in available protein-protein interaction data. Here we use the information coming from ligand-binding experiments to obtain “negative data”, i.e., non-protein-binding proteins: considering the inaccuracies in the protein-protein interaction data, if a protein has no experimental evidence of binding with another protein, but with a ligand, then we assume that the protein is non-protein binding. Our hypothesis here is that if a protein interacts with a ligand and no experimental data are available for its interaction with another protein, then the lack of evidence of protein-protein interaction is less likely due to the incompleteness in the data and more likely due to the lack of protein binding activity. Thus, we assembled a set of ligand-binding proteins and filtered out those that had high sequence similarity to proteins known to bind with other proteins to obtain a set of non-protein binding proteins. The methodology (described in detail in the Methods section) is not without its drawbacks: it disregards ligand-interacting proteins that are also involved in protein-protein interactions *in vivo* but lacking the confirmation of *in vitro* experimental data.

**Figure 4:**
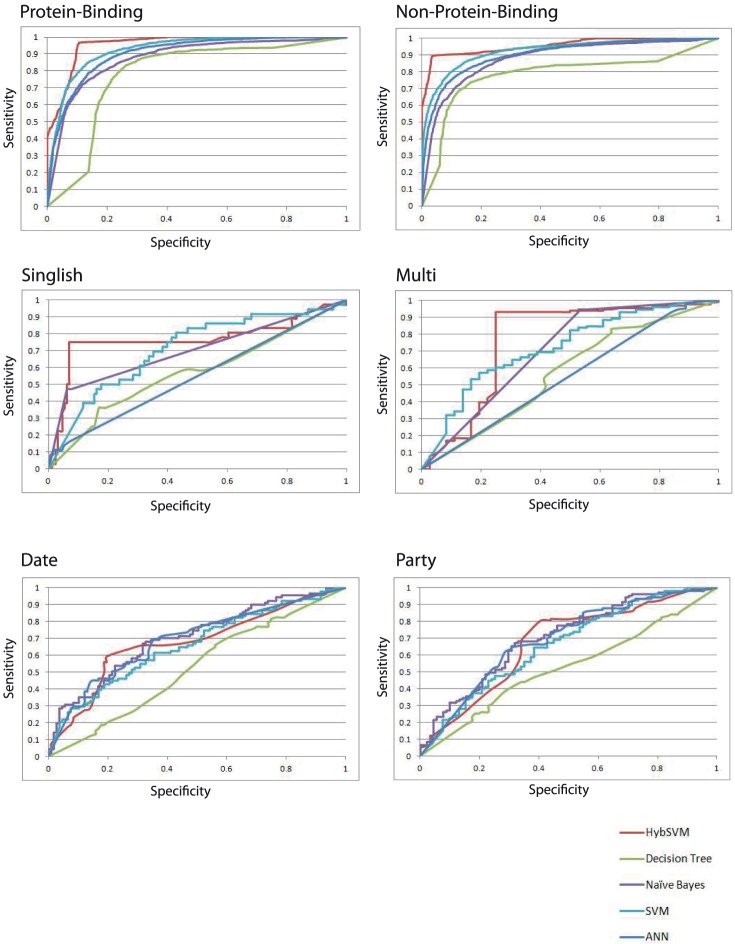
Receiver-operator characteristics (ROC) curve for Datasets 1, 3, and 4. The curve describes the tradeoff between sensitivity and specificity at different thresholds for various predictors. A simple domain-based method is included as a baseline for comparison. The figure includes ROC curves for protein-binding (PB) versus non-protein-binding (NPB), singlish-interface versus multi-interface hub proteins, and date versus party hub proteins.

As shown in Tables S1 and S2, the ability to distinguish protein-binding proteins from non-protein-binding proteins varies as a function of the machine learning method used and the size of the *k*-gram used. The accuracies ranged from 74.4% (Decision Tree, *k* = 2) to 87.2% (SVM, *k* = 2). Simply predicting each protein as belonging to the majority class yields an accuracy of 68.2% (see Domain-based method). Most of the methods were able to achieve accuracies well above 68.2%. The precision values ranged from 0% to 81%, recall from 0% to 93%, and correlation coefficient from 0.00 to 0.69. Figure 4 shows ROC curves for each of the methods. These curves show no single method outperforms all others over the entire range of tradeoffs between precision and recall. This suggests the possibility of using an ensemble of classifiers that takes advantage of the complementary information provided by the individual classifiers.

To examine this possibility, we built *HybSVM* for Phase I, which constructs a support vector machine (SVM) classifier that takes as input, for each protein sequence to be classified, the outputs of seven classifiers as well as the PSI-BLAST method and produces as output, a class label for the protein. The 7 classifiers used are: NB(1), NB(2), NB(3), NB(4), NB 2-gram, NB 3-gram, NB 4-gram. PSI-BLAST performs well on sequences with high sequence similarity whereas the NB(k) and NB k-gram methods perform well on sequences with high *k-*gram composition similarity. Logistic regression models are applied to the *HybSVM* classifier to get a probability score for each prediction. These scores are then used to evaluate the quality of each prediction.

Table 3 compares the performance of the *HybSVM* classifiers for Phase I against other standard machine learning approaches. *HybSVM* had an accuracy of 94.2% (an improvement of 6% in absolute terms over NB 4-gram) and a correlation coefficient of 0.87 (an improvement of 0.15 over NB 4-gram). For each performance measure the *HybSVM* method had the highest value for Dataset 1. *HybSVM* for Phase I also outperforms the other methods over the entire range of tradeoffs between precision and recall on a ROC curve (Figure 4).

**Table 3:** Dataset 1 (protein-binding vs. non-protein-binding, i.e. PB vs. NPB) prediction results from classifiers trained using machine learning methods.

Approach	Best k	Accuracy	F1 Score	Precision	Recall	C.C.	AUC
NB k-gram	4	88.2	80.1	.75	.86	.72	.85
NB(k)	3	86.4	78.5	.79	.78	.69	.83
Decision Tree	1	81.6	69.9	.72	.68	.57	.78
SVM	2	87.2	78.9	.82	.76	.70	.84
ANN	2	86.9	77.6	.83	.73	.71	.84
Naive Bayes	2	82.1	72.9	.70	.76	.60	.88
Domain-based	N/A	68.2	0.0	.00	.00	.00	.50
Homology-based	N/A	52.7	49.1	.37	.73	.15	N/A
HybSVM	N/A	**94.2**	**90.5**	**.92**	**.89**	**.87**	**.93**

Accuracy, F-measure (F1 Score), precision, recall, correlation coefficient (C.C.), and area under the receiver operating characteristic curve (AUC) of classification for the multi-interface versus singlish-interface dataset are presented. Accuracy and F-measure are reported in percentage. For each machine learning approach, values of k ranged from 1 to 4. Only the classifier with the best performing k-value (as defined by highest correlation coefficient) is shown. Our methods were estimated by cross-validation. The highest performing value(s) for each performance measure is highlighted in bold.

### Predicting hub proteins (Phase II)

Since our overall goal is to predict structural and kinetic classes for hub proteins and these classifiers need to be trained on hub-only proteins, we need a method to (1) identify hub proteins, (2) filter out non-hub proteins, and/or (3) flag proteins that have potential of being non-hubs. To evaluate this type of method, we assembled Dataset 2, consisting of 4,036 proteins including 1,741 hub proteins and 2,295 non-hub proteins. The dataset was derived from high confidence protein-protein interaction data from BioGrid [50] by labelling proteins with more than 5 interaction partners as hubs and proteins with fewer than 3 interaction partners as non-hubs. Proteins with 3, 4, or 5 interaction partners were not used in the dataset because, given the incompleteness of experimentally determined interactions, their categorization into hubs versus non-hubs is likely to be less reliable than the rest of the proteins in the dataset.

We used a simple homology-based method to classify proteins into hubs and non-hubs. A protein is classified as a hub if each of the top 4 hits returned by PSI-BLAST [51] search correspond to hub proteins (See Methods for details). Similarly, a protein is classified as a non-hub if all of the top hits are non-hub proteins. A protein is flagged as being likely a hub or non-hub based on the majority of the class-labels of the four top hits. If no hits are reported, the protein is flagged as having no known label. In addition to our predictions, in our web server, we report the number of interaction partners belonging to the top hit, the range of interaction partners of the top four hits, and a predicted number of interaction partners (based on the number of interaction partners of the top four BLAST hits weighted by the BLAST score of each hit). This simple sequence-based method correctly classified 536 hub proteins and 630 non-hub proteins (approximately 30% of the data). No proteins were incorrectly classified as hubs or non-hubs.

### Predicting structural and kinetic classes for hub proteins (Phase III)

#### Structural prediction: discriminating SIH from MIH hub proteins

To evaluate structural predictions on hub proteins, we created Dataset 3. The dataset consists of 155 hub proteins including 35 SIH and 120 MIH. The dataset is a subset of data originally compiled by Kim et al. [16], but has been filtered to remove highly homologous sequences (50% or more sequence identity within at least 80% of the length of the sequence).

Tables S3 and S4 show the ability to distinguish SIH and MIH (Dataset 3) based on several standard machine learning approaches with varying sizes *k*-grams. The accuracies ranged from 67.7% (Decision Tree, *k* = 2) to 81.2% (Naive Bayes, *k* = 3). Several classifiers actually had accuracies below 77.4% (e.g., SVM, *k* = 1). The precision values ranged from 0% to 86%, recall from 0% to 63%, and correlation coefficient from 0.00 to 0.41. Figure 4 shows ROC curves for each of the methods. Again, these curves show no single method outperforms all others. On Dataset 3, each of the machine learning methods used here outperformed the simple domain-based method (note that the simple domain-based method had both 0.00 precision and recall because it was unable to predict any SIH proteins correctly).

To validate how well interaction sites of SIH and MIH can be predicted on Dataset 3, we ran a subset of the data through the interaction site predictor ISIS [52] and the target specific interaction site predictor NPS-HomPPI [53] with default settings. Both methods generally under-predicted the number of interaction sites and in many cases the methods predicted few or no interaction sites on the hub proteins.

Table 4 shows that the individual methods perform well on assigning hubs to classes based on structural characteristics. No single *k-*value is optimal for all methods; optimal values of *k* vary with the size and complexity of the dataset. Variables such as number of proteins, size of proteins, and homology between proteins all play an important role in developing an appropriate model for our classifiers. Therefore, it is difficult to design a single model or choose a single optimal value of *k* for any dataset without prior knowledge of the data. We also observe that proteins within a class are assigned different labels by classifiers that correspond to different choices of *k*. Our results show that a single classifier does not classify all the proteins correctly, yet a vast majority of the proteins (over 93%) have at least one classifier that correctly predicts its class. Again, we used the *HybSVM* method, this time for Phase III classifications, to take advantage of the complementary information provided by the individual classifiers.

**Table 4:** Dataset 3 (SIH vs. MIH) prediction results from classifiers trained using machine learning methods.

Approach	Best k	Accuracy	F1 Score	Precision	Recall	C.C.	AUC
NB k-gram	4	83.8	53.8	.42	.75	.47	.71
NB(k)	3	83.2	45.3	.31	**.84**	.44	.69
Decision Tree	3	71.0	40.3	.38	.43	.21	.57
SVM	2	76.1	41.0	.46	.37	.27	.62
ANN	2	79.0	14.1	.60	.08	.10	.55
Naive Bayes	3	81.2	52.8	.62	.46	.41	.70
Domain-based	N/A	76.4	0.0	.00	.00	−.01	.42
Homology-based	N/A	66.4	46.6	.74	.34	.32	N/A
HybSVM	N/A	**89.0**	**76.0**	**.75**	.77	**.69**	**.85**

Accuracy, F-measure (F1 Score), precision, recall, correlation coefficient (C.C.), and area under the receiver operating characteristic curve (AUC) of classification for the multi-interface versus singlish-interface dataset are presented. Accuracy and F-measure are reported in percentage. For each machine learning approach, values of k ranged from 1 to 4. Only the classifier with the best performing k-value (as defined by highest correlation coefficient) is shown. Our methods were estimated by cross-validation. The highest performing value(s) for each performance measure is highlighted in bold.

From the results shown in Table 4, we can see that *HybSVM* outperforms all other individual methods on 5 of the 6 performance measures. For Dataset 3, *HybSVM* improved accuracy by 5.2% (89.0%) and correlation coefficient by 0.22 (0.69) over the previous best classifier, NB 4-gram. This method also had the highest AUC with a value of 0.85 (an improvement of 0.14 over SVM, the next highest performing method). NB(k) had the highest recall value at 0.84 (it was able to correctly label more SIH proteins), but it came at the cost of a low precision (0.31) and lower correlation coefficient (0.44). The threshold can be adjusted for *HybSVM* to achieve a better recall based on *HybSVM* having very balanced precision and recall scores (values of 0.75 and 0.77) and the highest f-measure (76.0). Figure 4 shows ROC curves for each of the methods on Dataset 3. Although *HybSVM* did not always outperform the other methods over the entire range of tradeoffs between precision and recall, it did outperform the other methods for a specific range of false positive rates (from 0.0 to 0.4 for SIH and from 0.25 to 0.5 MIH). No single method significantly outperformed *HybSVM.* It is worth noting that *HybSVM* method is especially attractive if there is little tolerance for false positives. In contrast, each of the other methods (with the exception of domain-based method) works relatively well, in settings where there is greater tolerance for higher false positive rates.

A closer examination of the results for Dataset 3 shows that many of the misclassified hub proteins are close to the arbitrary boundary between SIH and MIH. This raises the question as to whether the labels could be more reliably predicted if the arbitrary cut-off on the number of interfaces is altered (See Figure S1). For example, hubs with 4 or fewer interaction sites were labelled with an accuracy of 72%. However, the accuracy of classification of hubs with 3 or fewer interfaces, the cut-off value for distinguishing SIH and MIH, was considerably lower. The sensitivity of predictions for MIH improves as the number of interfaces of the hub protein increases (See Figure S2). The sensitivity of predicting a protein hub with four or more interfaces is 96% (119/124) and 97% (108/111) for protein hubs with 5 or more interfaces. The majority (11/18) of our misclassifications were caused by a strong homology between proteins with differing numbers of interaction partners that were 2 or fewer (e.g. a protein with two interaction partners had a strong homology with a protein that had four interaction partners). Details of the misclassifications can be found in Table 5.

**Table 5:** Details for misclassified proteins in Dataset 3 using HybSVM.

	Misclassified proteins		Protein with highest homology			
Class	Gene	Interfaces	Gene	Interfaces	e-value	Difference
Singlish	RHO1	3	CDC42	2	1E-52	1
singlish	STE11	4	CLA4	2	2E-37	2
singlish	ARP2	3	ACT1	2	1E-102	1
singlish	MAK5	9	DRS1	2	6E-42	7
singlish	PRP28	8	DRS1	2	2E-49	6
singlish	PUB1	3	SGN1	1	1E-05	2
singlish	CMD1	4	MLC1	2	1E-15	2
singlish	SEC22	7	YKT6	2	9E-10	5
singlish	SNP1	3	SGN1	1	8E-05	2
multi	YKT6	2	SEC22	7	9E-05	−5
multi	CDC42	2	RHO1	3	1E-05	−1
multi	ACT1	2	ARP2	3	1E-05	−1
multi	SGN1	1	PUB1	3	1E-05	−2
multi	YTA7	1	RPT4	6	8E-05	−5
multi	MLC1	2	CMD1	4	1E-05	−2
multi	MTR3	1	(No hit)	N/A	N/A	N/A
multi	BOI2	2	(No hit)	N/A	N/A	N/A
multi	CLA4	2	STE11	4	2E-05	−2

Details for the misclassified proteins in Dataset 3 based on using the *HybSVM* method including: actual class (multi, singlish), gene name, and number of interfaces as predicted by Kim et al. [16] are shown. For each misclassified protein, information about the protein with the highest homology based on the nearest BLAST hit is also shown. This information includes: gene name, number of interfaces as predicted by Kim et al. [16], e-value of the BLAST results between the two proteins, and the difference between the number of predicted interfaces for the misclassified protein and its nearest BLAST hit.

#### Kinetic prediction: discriminating Date from Party hub proteins

To assess kinetic predictions on hub proteins, we created Dataset 4. Dataset 4 contains 199 hub proteins including 91 date hubs and 108 party hubs. Dataset 4 was originally created by Han et al. [31]; this dataset had relatively low sequence homology so no sequences were removed. Tables S5 and S6 show the results of using standard machine learning approaches on this dataset. The accuracies for Dataset 4 ranged from 51.0% to 66.2% and the correlation coefficients from 0.01 to 0.30; precision from 50% to 70% and recall from 42% to 62%. The results of HybSVM on Dataset 4 (see Table 6) provided an accuracy of 69.2% and a correlation coefficient of 0.37, which are comparatively better than the best individual method (NB 3-gram). HybSVM had a marginally lower AUC value (0.68) as compared to the Naive Bayes (0.70). HybSVM also had the best F-Score (62.6) and precision (0.71). Figure 4 shows ROC curves for each of the methods on Dataset 4. The ROC curves were similar to the curves generated by building classifiers on the SIH/MIH dataset. The results show that *HybSVM* method can be used in settings where a low false positive rate is desirable.

**Table 6:** Dataset 4 (Date vs. Party hubs) predictions from classifiers trained using machine learning methods.

Approach	Best k	Accuracy	F1 Score	Precision	Recall	C.C.	AUC
NB k-gram	3	67.1	59.8	.54	**.67**	.33	**.71**
NB(k)	3	65.1	58.0	.53	.64	.29	.65
Decision Tree	1	53.5	55.4	.50	.62	.08	.53
SVM	3	62.1	59.0	.59	.59	.24	.66
ANN	2	66.2	55.5	.70	.46	.30	.69
Naive Bayes	1	65.2	57.5	.66	.51	.29	.70
Domain-based	N/A	59.1	30.2	.62	.20	.14	.57
Homology-based	N/A	29.8	22.0	.22	.22	−.43	N/A
HybSVM	N/A	**69.2**	**62.6**	**.71**	.56	**.37**	.68

Accuracy, F-measure (F1 Score), precision, recall, correlation coefficient (C.C.), and area under the receiver operating characteristic curve (AUC) of classification for the multi-interface versus singlish-interface dataset are presented. Accuracy and F-measure are reported in percentage. For each machine learning approach, values of k ranged from 1 to 4. Only the classifier with the best performing k-value (as defined by highest correlation coefficient) is shown. Our methods were estimated by cross-validation. The highest performing value(s) for each performance measure is highlighted in bold.

A study by Mirzarezaee et al. has recently proposed methods for distinguishing date hub proteins from party hub proteins [15] using a variety of features including 4 composition measurements, 48 physicochemical properties, six GO term features, domain occurrence presence, disordered regions, and position specific scoring matrices (PSSM). They reported accuracies of up to 77% with correlation coefficients of 0.74 for both date and party hubs. Their dataset also used yeast proteins, but it was a different set of proteins and consisted of over 5,000 non-hub proteins. Their methodology consisted of classifying proteins into the following four classes: non-hub, intermediately connected, date, and party. The *HybSVM* approach we report here focused instead on the binary classification task of distinguishing date hubs from party hubs. Our method does not need functional annotations (GO terms) of proteins, their domain composition, or their sequence alignments with their homologs. Our method also provides probability scores for each prediction. These scores allow an investigator to trade-off the reliability of predictions against the coverage of the predictions. *HybSVM* runs quickly and is easy to implement and update, which are ideal characteristics to serve the method through a web server. A web server implementation of *HybSVM* can be found here: http://hybsvm.gdcb.iastate.edu.

### Validating the three-phase approach

To validate our three-phase approach, we tested each phase on additional datasets. Since Dataset 1 (PB versus NPB) was created independently of Dataset 3 (SIH versus MIH) and Dataset 4 (Date versus Party), we used these two datasets along with the data used in the Mirzarezaee paper (Date versus Party) as a test set for the *HybSVM* classifier for predicting protein-binding proteins (Phase I). The union of these three datasets included 900 yeast hub proteins. The Phase I *HybSVM* classifier predicted 99.7% of the proteins as protein-binding proteins. Only three multi-interface proteins were misclassified.

We also used the data from the Mirzarezaee study as an additional test set to predict hub proteins (Phase II) and for the *HybSVM* classifier to discriminate date hubs from party hubs (Phase III). The Phase II classifier correctly predicted 147 proteins as hub proteins and 116 as likely hub-proteins. The classifier misclassified 45 proteins as non-hub proteins and 23 as likely non-hubs proteins (12% error rate). All other proteins were labeled as being of unknown category. The Phase III classifier used to discriminate date and party hub proteins predicted 67.9% of the 546 proteins correctly with a correlation coefficient of 0.36. One of the advantages of our approach over the Mirzarezaee study is that a probability score is assigned to the predictions. In this example, a majority of the misclassifications had a probability score under 0.70. Predictions with higher scores are more reliable. For example, in the case of predictions with score greater than 0.70 (337 proteins), accuracy improves to 74.2% (0.46 correlation coefficient). The predictions with score greater than 0.90 (78 proteins) yield even more reliable results: 84.6% accuracy, and 0.54 correlation coefficient. These results show that investigators can benefit from our method, which needs only sequence information, to control the quality of the predictions by sacrificing the coverage of the classifier. SIH and MIH class labels were not readily available for the Mirzarezaee dataset, so the structural classifier of Phase III was not evaluated on this dataset.

### Conclusion

We have demonstrated that it is possible to fairly reliably classify proteins in a three-phase approach: the first phase distinguishes protein-binding (PB) versus non-protein-binding (NPB) proteins; the second phase predicts if the protein is likely to be a hub; the third phase classifies protein-binding proteins into SIH versus MIH and date versus party hubs. Our approach uses only sequence information and therefore will be highly useful for the analysis of proteins lacking structural information. These classifications provide insights into the structural and kinetic characteristics of the corresponding proteins in the absence of interaction networks, expression data, three-dimensional structure, sequence alignment, functional annotations, domains, or motifs. We note that the performance of our classifier for predicting structural characteristics of hubs (i.e., classifying hubs into SIH versus MIH) is better than that of the classifier for predicting kinetic or expression related characteristics of hubs (i.e., classifying hubs into date versus party hubs).

## Materials and Methods

Here we used four datasets for training and testing classifiers for different phases of prediction. Because protein interaction datasets tend to have high false positive rates, when building these datasets, our main goal was to use high-quality data. Our second goal was to remove sequence bias in the datasets. The first dataset consists of proteins that are involved in binding with other proteins (PB) and proteins that are not involved in PB (NPB). This dataset was used in the first phase of our prediction. The second dataset consists of hub and non-hub proteins. This set of proteins was used in the second phase of predictions. Datasets 3 and 4 were used by the third phase to distinguish singlish/multiple and date/party hub proteins (Figure 2).

### Dataset 1 – Protein-binding (PB) versus non-protein-binding (NPB) proteins

The first dataset consists of two subsets of proteins. The first subset is generated using high-quality sets of proteins that are known to interact with other proteins. These proteins form a protein-binding (PB) subset. The second subset consists of proteins that are unlikely to bind with other proteins (NPB). To create the PB subset, 3,640 yeast proteins were downloaded from HINT [9] (High-quality protein interactomes) (http://hint.yulab.org/)–HINT is a database of high-quality protein-protein interactions for different organisms, which was obtained by integrating protein-protein interaction data from various sources and filtered to remove low-quality interactions.

The NPB subset consists of proteins that bind with small molecules, but not with proteins. Identifying such a subset is a challenging task, because the available protein-protein interaction data are incomplete at best. It has been estimated that the fraction of identified interactions of the full human interactome is between 5% and 13% [8], [54], [55] and up to 30% for the yeast interactome [54]. The efforts to increase the coverage will most likely increase the false positive rate as well [56]. Therefore, it is inevitable that any NPB dataset will be subject to these inherent limitations of incompleteness and incorrectness in experimental protein-protein interaction sets. Considering these limitations, we used the following methodology to create the NPB subset: a set of 8,443 proteins were downloaded from BindingDB [57] (http://www.bindingdb.org/bind/index.jsp). This includes the entire set of protein targets that bind to small-molecules. In order to filter proteins that are interacting with other proteins, these 8,443 BindingDB proteins were BLASTed [58] against the PB set and any protein that had a positive hit was removed. Additional filtering was performed with the remaining BindingDB proteins against the 5,000 yeast proteins that have an experimental protein-protein interaction evidence in BioGrid [50] (http://thebiogrid.org/). The remaining set of non-interacting proteins was 4,567 proteins. To minimize sequence bias, we clustered the proteins in both subsets where at least 80% of the sequence shared 50% or more sequence identity. A representative sequence was randomly chosen for each cluster to obtain the final dataset. The resulting dataset, Dataset 1, consists of a total of 5,010 proteins including 3,418 proteins in the PB subset and 1,592 proteins in the NPB subset.

### Dataset 2 – Hub proteins versus non-hub proteins

Manna et al. [18] had previously created a dataset of hubs and non-hubs. This dataset was originally assembled by downloading human protein-protein interaction data from BioGRID [50]. Any protein that had more than five interactions was labeled as a hub, proteins with fewer than three interactions were labeled as non-hub. Proteins with three, four, or five interactions were not considered as they were near the arbitrary cut-off value for defining a hub and had high potential for being mislabeled. Their resulting dataset included 2,221 hub proteins and 2,889 non-hub proteins. The data ranged from proteins with a single interaction partner to 170 interaction partners. To minimize sequence bias in this dataset, we applied the same methodology we used to obtain Dataset 1: we clustered the protein where at least 80% of each sequence shared 50% or more sequence identity and randomly chose a representative sequence from each cluster. The resulting dataset, Dataset 2, consists of 4,036 proteins including 1,741 hub proteins and 2,295 non-hub proteins.

### Dataset 3 - Singlish interface hubs (SIH) versus multiple interface hubs (MIH)

Previously Kim et al. [16] created SIH and MIH datasets by combining yeast interaction data from various sources [4], [16], [59]–[63] and associating these proteins with Pfam domains [49], [64], which were then subsequently mapped onto known PDB structures using iPfam [65]. They filtered out interactions that were not consistent with protein complexes as defined by iPfam to obtain a yeast protein interaction network. Kim et al. used this set to analyze evolutionary patterns in hub proteins and it uses a robust structure-based definition of hubs, which was useful in our study. Here, we follow their definition of hubs such that a protein is defined as a hub protein if it has five or more interaction partners. A hub protein with 1 or 2 mutually exclusive interactions is defined as singlish-interface hub (SIH); a hub protein with 3 or more mutually exclusive interactions is defined as multi-interface hub (MIH). The original dataset consists of 1,269 interactions involving 873 proteins with 167 hub proteins including 36 SIH and 131 MIH proteins. We downloaded the original dataset from http://sin.gersteinlab.org. We filtered non-hub proteins out and applied the same sequence filtering that we used for Dataset 1 and Dataset 2. The resulting dataset, Dataset 3, consists of 155 hub proteins with 35 SIH and 120 MIH proteins.

### Dataset 4 - Date hubs versus Party hubs

Han et al. [31] created a protein set of date and party hubs by merging the results of multiple methods [4], [31], [32], [59]–[62], [66]–[71]. Similar to Kim et al. [16], they defined a protein as a hub protein if it has five or more interaction partners. They based their definition of date and party hubs on co-expression patterns: hubs that have low degree of co-expression with their interaction partners (Pearson correlation coefficient of 0.5 or lower) are assumed to bind different partners at different time points or locations and are classified as date hubs. In the same vein, hubs that exhibit high degree of co-expression with their interaction partners (Pearson correlation coefficient greater than 0.5) are assumed to interact simultaneously with their interaction partners. Their resulting yeast interaction dataset consists of 1,379 proteins and 2,493 interactions, which contains both hub and non-hub proteins. We filtered non-hub proteins out and applied the same sequence filtering that we used on the previous datasets. The sequence bias was already removed in the original dataset; so no additional sequences were removed. The resulting set, Dataset 4 consists of 199 hub proteins including 91 date hubs and 108 party hubs.

### Overlap among Dataset 3 and Dataset 4

**Figure 5:**
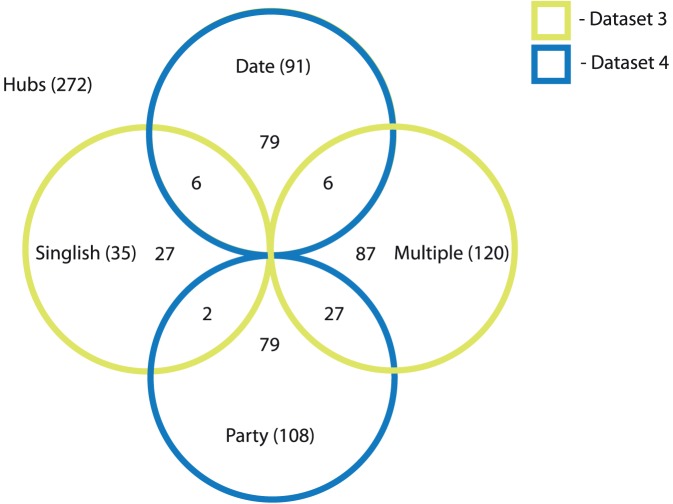
Venn-diagram for Dataset 3 and Dataset 4. Each of the 272 hub proteins belong to one or more of the following classes: singlish, multi, date, party. Dataset 3 consists of 35 singlish hub proteins and 120 multi hub proteins (Yellow circles). Dataset 4 consists of 91 date hub proteins and 108 party hub proteins (Blue circles). Please see text for more details about the datasets.

**Figure 6:**
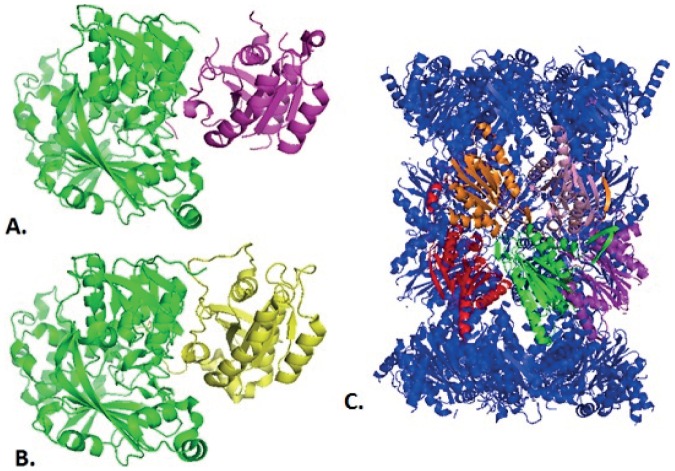
Example of a singlish-interface date and multi-party hub proteins. Images A and B show the quaternary structure for the singlish-date protein Rab GDP dissociation inhibitor alpha (GDI1, YER136W) binding with two different proteins. Image C shows the quaternary structure for the yeast protein beta 6 subunit of the 20S proteasome (PRE7, YBL041W) binding with multiple proteins at the same time. **A:** GDI1 (green) binding with GTP-binding protein YPT31/YPT8 (purple). PDB ID of the complex: 3cpj [79], [80]. **B:** GDI1 (green) binding with GTP-binding proteinYPT1 (yellow). PDB ID of the complex: 1ukv [80], [81]. The protein binds at one location (singlish-interface) with one partner at a time (date). **C:** PRE7 (green) binds with PUP1 (orange), PUP3 (red), C5 (pink), PRE4 (purple). PDB ID of the complex: 3bdm [80], [82]. The protein binds at multiple locations (multi-interface) with many partners at same time (party).

It is worth noting that the SIH-MIH and Date-Party classes show some overlap. Figure 5 is a Venn diagram showing the distribution of the 41 proteins in their respective classes. For example, 6 singlish-interface proteins are also date hub proteins. Similarly, there are 2 singlish-party hubs, 6 multi-date hubs, and 27 multi-party hubs. Figure 6 show examples of singlish-interface date hub and multi-party hub proteins respectively.

### Classification framework

For each class within our dataset we built a binary classifier that predicts if that protein belonged to that class or not. The reported accuracy estimates are based on stratified 10-fold cross validation. Each of the individual classifiers is described below:

### Machine learning methods

#### Support Vector Machines

A support vector machine (SVM), given a training set, that is linearly separable in a kernel-induced feature space, implements a linear decision boundary that maximizes the margin of separation between the classes [46]. If the dataset is not perfectly separable, slack variables are used to minimize the number of misclassified training examples. Logistic regression models were applied to the outputs of the SVM to get a probability score. These scores can be used to evaluate the quality of a prediction. Even if the overall accuracy of the prediction model does not meet high standards, the quality of individual predictions may be suitable based on the probability score. The score also allows an investigator to determine and set the trade-off between the sensitivity and selectivity of the classified (as shown in the ROC-curves). The scores range from 0.5 (50% or equal probability of belonging to either class) to 1 (100% probability of belonging to the specified class). We used the Weka version 3.6.0 SMO implementation [45] of the support vector machine algorithm [46] with a polynomial kernel.

#### Naive Bayes Multinomial Classifier

The Naive Bayes multinomial classifier models each sequence by a bag of letters sampled from a fixed alphabet. In our case, the bag of letters is the amino acid composition of a protein sequence. Thus, each element (amino acid) of the sequence is assumed to be independent of the other elements in the sequence given the class label. Based on this assumption, a multinomial Naive Bayes classifier can be built over all of the sequences for a given class. This is similar to the bag of words approach previously used for text classification [72].

#### Naive Bayes k-grams Classifier

The Naive Bayes *k*-grams (NB *k*-grams) method [73] uses a sliding a window of size *k* along each sequence to generate a *bag* of *k*-grams representation of the sequence. Much like in the case of the Naive Bayes classifier described above, the Naïve Bayes *k*-grams classifier treats each *k*-gram in the bag to be independent of the others given the class label for the sequence. Given this probabilistic model, the previously outlined method for classification using a probabilistic model can be applied. A problem with the NB *k*-grams approach is that successive *k*-grams extracted from a sequence share *k*-1 elements in common. This grossly and systematically violates the independence assumption of Naive Bayes.

#### NB (k)

NB(k) [73] constructs, for each class, a Markov model of order *k* -1. It modifies the Naïve Bayes model to explicitly model the dependencies (of order *k-1*) between the letters of a sequence. It is easily seen that when *k* = 1, Naive Bayes k-grams as well as Naive Bayes (1) reduce to the Naive Bayes model. The relevant probabilities required for specifying the above models can be estimated using standard techniques for estimation of probabilities using Laplace estimators [74].

Naïve Bayes (NB) *k*-grams and NB(*k*) models were constructed and evaluated on the dataset with *k* ranging from 1 to 4. Values of *k* larger than 4 were not considered because at higher values of *k*, the available data are insufficient to obtain reliable probability estimates.

#### PSI-Blast

The homology-based tool PSI-BLAST [51] version 2.2.9 was used to construct a binary classifier for each class. We used the binary class label predicted by the PSI-BLAST-based classifier as an additional input to our *HybSVM* classifier. Given a query sequence to be classified, we used PSI-BLAST to compare the query sequence against the training set. In 10-fold cross-validation, we ran PSI-BLAST with the query sequences in a given fold against the reference database comprised of the remaining nine folds. We repeated this process for each of the ten folds. For *HybSVM*, a class was assigned to the query sequence based on the top-scoring hit (i.e., the sequence with the lowest e-value) from the PSI-BLAST results. The resulting binary prediction of the PSI-BLAST classifier for class *c* is 1 if the class label for the top scoring hit is *c*. Otherwise, it is 0. An e-value cut-off of 1×10^−4^ was used for PSI-BLAST, with all other parameters set to their default values. For predicting hub proteins, the four top-scoring hit were used. If there was a consensus among the top four hits then the class label of the four hits is assigned to the query sequence. If three of the four top-scoring hits had the same class, the query sequence is labeled as ‘likely’ belonging to that class. In addition to the prediction, we report the number of interaction partners belonging to the top hit, the range of interaction partners of the top four hits, and a weighted average (based on the BLAST score) of number of interaction partners of the top four hits.

#### Domain-based Method

The domain-based method builds a classifier by using a class-conditional probability distribution based on the frequency of SCOP [47], [48] and PFAM [49] domains in the following manner. For each protein, the count for each type of domain was determined by the number of domains listed at the Saccharomyces Genome Database (SGD) [75]. This method was used to rule out a simple direct correlation between the number of domains and the number of interaction sites on a hub protein.

#### Decision Tree

A Decision Tree builds a predictive model by recursively partitioning the dataset based on choosing features that provide the most information gain. In our example, the feature set is the observed k-gram composition of amino acids given a class. For binary classes (e.g., SIH versus MIH), the decision tree algorithm chooses a *k*-gram feature that partitions the data to maximize the information gain between classes. The process is recursively repeated on the new partitions until no more information gain can be achieved. Additional techniques are performed (e.g., pruning) to help prevent overtraining. For these experiments we used the commonly used decision tree algorithm C4.5 [44] implemented as the J4.8 algorithm [43] in Weka version 3.6.0.

#### Multi-layer perceptron

A multi-layer perceptron, often referred to as a multilayer artificial neural network [76], [77] (ANN) implements a non-linear decision function by using a weighted linear combination of non-linear (typically sigmoid) transformations of linear functions of input features. The ANN is typically trained using error back-propagation or generalized gradient descent algorithm that minimizes a function of the error between the desired and actual outputs of the ANN. We used the Multilayer Perceptron artificial neural network (ANN) implementation [43] in Weka version 3.6.0.

#### HybSVM Method

We introduce *HybSVM* classifier that is a support vector machine (SVM) classifier that assigns the class label to a target sequence based on the class labels output by the Naive Bayes (NB), NB k-gram, NB(k) classifiers, and an additional attribute, the output from the PSI-BLAST classifiers. Since there are eight classifiers Naive Bayes, NB 2-gram, NB 3-gram, NB 4-gram, NB(2), NB(3), NB(4), and PSI-BLAST, the input to the *HybSVM* classifier consists of a 8-tuple vector of class labels assigned to the sequence by the 8 classifiers. A SVM is trained to predict the class label for each sequence based on the 8-tuple of class labels predicted by the eight individual classifiers.

### Performance evaluation

The performance measures *accuracy, precision, recall, f-measure, and correlation coefficient* are used to evaluate the performance of our machine learning approaches [78]. *Accuracy* is the fraction of overall predictions that are correct. *Precision* is the ratio of predicted true positive examples to the total number of actual positive examples. *Recall* is the ratio of predicted true positives to the total number of examples predicted as positive. The *F-measure* (F1 score) is the harmonic mean of precision and recall. The F-measure has a range between 0 (worst value) and 1 (best value). *Correlation coefficient* measures the correlation between predictions and actual class labels. The correlation coefficient has a range of -1 (worst value) and 1 (best value).

Table S7 summarizes the definitions of performance measures in the two-class setting (binary classification), where *M* represents the total number of classes and *N* represents the total number of examples. *TP*, *TN*, *FP*, and *FN* are the true positives, true negatives, false positives, and false negatives for each of our classification problems. For example, when predicting SIH proteins: TP refers to a SIH correctly predicted, FP to MIH predicted as SIH, FN as SIH predicted as a MIH, and TN to MIH correctly predicted.

Where possible, we used the area under the receiver operating characteristic (AUC) curve. The ROC curve plots the true positive rate versus false positive rate for a binary classifier system (a protein belongs to a given class or not) as its discrimination threshold is varied. The area ranges from 0 (worst) to 1 (best); the value of 0.5 refers to the expected value of a random method.

## Supporting Information

Figure S1 **The accuracy curve of predicting singlish-interface and multiple-interface hub proteins as a function of the number of interaction sites.** The curve shows the prediction accuracy for proteins with number of interactions sites less than the given maximum threshold. For example, the value of 5 on the x-axis refers to all hub proteins with 5 or fewer interfaces and the value on the curve (83%) at x = 5, represents the accuracy of this set.
(DOCX)

Figure S2 **The sensitivity curve of predicting singlish-interface and multiple-interface hub proteins as a function of the number of interaction sites.** The curve shows the prediction accuracy for proteins with number of interactions sites more than the given minimum threshold. For example, the value of 5 on the x-axis refers to all hub proteins with 5 or more interfaces and the value on the curve (97%) at x = 5, represents the sensitivity of this set.
(DOCX)

Table S1 **Accuracy, precision, recall, and correlation coefficient (CC) of classification for the protein-binding versus non-protein-binding dataset are presented for internal machine learning methods.** For each machine learning approach, values of k ranged from 1 to 4. The performance of the results were estimated using cross-validation. The highest performing value(s) for each performance measure is highlighted in bold.
(DOCX)

Table S2 **Accuracy, precision, recall, and correlation coefficient (CC) of classification for the protein-binding versus non-protein-binding dataset are presented for standard machine learning methods.** For each machine learning approach, values of k ranged from 1 to 2. The performances of the results were estimated using cross-validation. The highest performing value(s) for each performance measure is highlighted in bold.
(DOCX)

Table S3 **Accuracy, precision, recall, and correlation coefficient (CC) of classification for the multi-interface versus singlish-interface dataset are presented for internal machine learning methods.** For each machine learning approach, values of k ranged from 1 to 4. The performances of the results were estimated using cross-validation. The highest performing value(s) for each performance measure is highlighted in bold.
(DOCX)

Table S4 **Accuracy, precision, recall, and correlation coefficient (CC) of classification for the multi-interface versus singlish-interface dataset are presented for standard machine learning methods.** For each machine learning approach, values of k ranged from 1 to 3. The performances of the results were estimated using cross-validation. The highest performing value(s) for each performance measure is highlighted in bold.
(DOCX)

Table S5 **Accuracy, precision, recall, and correlation coefficient (CC) of classification for the date versus party dataset are presented for internal machine learning methods.** For each machine learning approach, values of k ranged from 1 to 4. The performances of the results were estimated using cross-validation. The highest performing value(s) for each performance measure is highlighted in bold.
(DOCX)

Table S6 **Accuracy, precision, recall, and correlation coefficient (CC) of classification for the date versus party dataset are presented for standard machine learning methods.** For each machine learning approach, values of k ranged from 1 to 3. The performances of the results were estimated using cross-validation. The highest performing value(s) for each performance measure is highlighted in bold.
(DOCX)

Table S7 **The formula for binary classification for each of our five performance measures is provided.** ***TP*** **,** ***TN*** **,** ***FP*** **,** ***FN*** **are the true positives, true negatives, false positives, and false negative predictions.**
(DOCX)
