# Supplementary material for: Predicting the Binding Patterns of Hub Proteins: A Study Using Yeast Protein Interaction Networks
Source: PLoS One. 2013 Feb 19;8(2):e56833. doi: 10.1371/journal.pone.0056833 (PMC3576370; doi:10.1371/journal.pone.0056833)
Supplement: Figure S1 — The accuracy curve of predicting singlish-interface and multiple-interface hub proteins as a function of the number of interaction sites. The curve shows the prediction accuracy for proteins with number of interactions sites less than the given maximum threshold. For example, the value of 5 on the x-axis refers to all hub proteins with 5 or fewer interfaces and the value on the curve (83%) at x = 5, represents the accuracy of this set. (DOCX) [file pone.0056833.s001.docx]

**
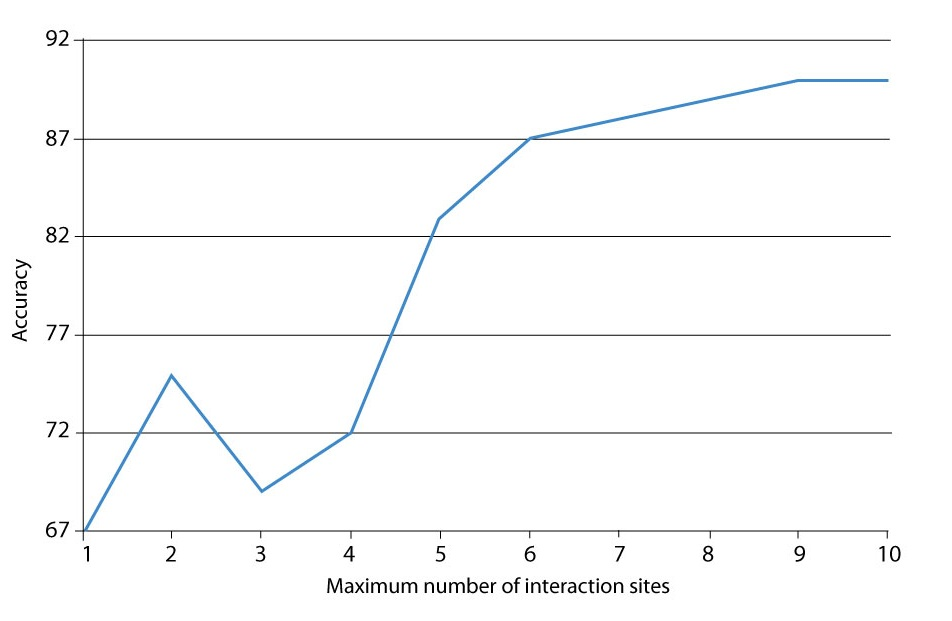
**

**Figure S1. The accuracy curve of predicting singlish-interface and multiple-interface hub proteins as a function of the number of interaction sites.** The curve shows the prediction accuracy for proteins with number of interactions sites less than the given maximum threshold. For example, the value of 5 on the x-axis refers to all hub proteins with 5 or fewer interfaces and the value on the curve (83%) at x=5, represents the accuracy of this set.
