# Supplementary material for: Predicting the Binding Patterns of Hub Proteins: A Study Using Yeast Protein Interaction Networks
Source: PLoS One. 2013 Feb 19;8(2):e56833. doi: 10.1371/journal.pone.0056833 (PMC3576370; doi:10.1371/journal.pone.0056833)
Supplement: Figure S2 — The sensitivity curve of predicting singlish-interface and multiple-interface hub proteins as a function of the number of interaction sites. The curve shows the prediction accuracy for proteins with number of interactions sites more than the given minimum threshold. For example, the value of 5 on the x-axis refers to all hub proteins with 5 or more interfaces and the value on the curve (97%) at x = 5, represents the sensitivity of this set. (DOCX) [file pone.0056833.s002.docx]

**
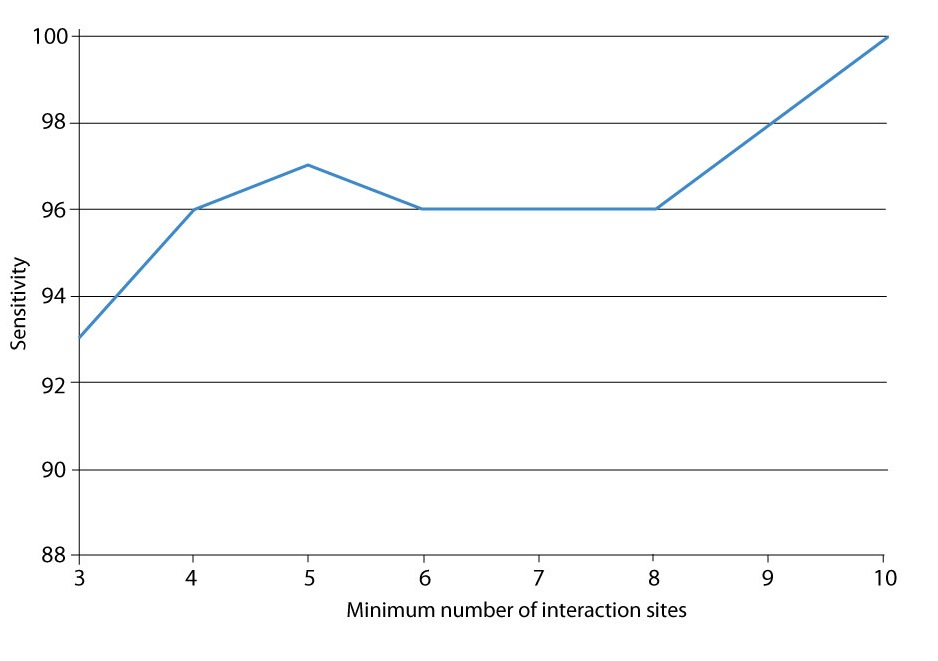
**

**Figure S2**. **The sensitivity curve of predicting singlish-interface and multiple-interface hub proteins as a function of the number of interaction sites.** The curve shows the prediction accuracy for proteins with number of interactions sites more than the given minimum threshold. For example, the value of 5 on the x-axis refers to all hub proteins with 5 or more interfaces and the value on the curve (97%) at x=5, represents the sensitivity of this set.
