# Supplementary material for: Predicting the Binding Patterns of Hub Proteins: A Study Using Yeast Protein Interaction Networks
Source: PLoS One. 2013 Feb 19;8(2):e56833. doi: 10.1371/journal.pone.0056833 (PMC3576370; doi:10.1371/journal.pone.0056833)
Supplement: Table S1 — Accuracy, precision, recall, and correlation coefficient (CC) of classification for the protein-binding versus non-protein-binding dataset are presented for internal machine learning methods. For each machine learning approach, values of k ranged from 1 to 4. The performance of the results were estimated using cross-validation. The highest performing value(s) for each performance measure is highlighted in bold. (DOCX) [file pone.0056833.s003.docx]

**Table S1.** Dataset 1 results on our internal machine learning methods. Accuracy, precision, recall, and correlation coefficient (CC) of classification for the protein-binding versus non-protein-binding dataset are presented for internal machine learning methods. For each machine learning approach, values of k ranged from 1 to 4. The performances of the results were estimated using cross-validation. The highest performing value(s) for each performance measure is highlighted in bold. NB stands for Naïve Bayes.

| Approach | k | Accuracy | Precision | Recall | CC |
| --- | --- | --- | --- | --- | --- |
| NB k-gram | 1 | 81.4 | .78 | .68 | .59 |
|  | 2 | 82.5 | .80 | .69 | .61 |
|  | 3 | 84.5 | .81 | .72 | .65 |
|  | 4 | 88.2 | .75 | .86 | .72 |
| NB(k) | 2 | 83.9 | .80 | .72 | .64 |
|  | 3 | 86.4 | .79 | .78 | .69 |
|  | 4 | 85.8 | .59 | **.93** | .66 |
| Domain-based | N/A | 68.2 | .00 | .00 | .00 |
| Homology-based | N/A | 52.7 | .37 | .73 | .15 |
| **HybSVM** | **N/A** | **94.2** | **.92** | .89 | **.87** |
