# Supplementary material for: Predicting the Binding Patterns of Hub Proteins: A Study Using Yeast Protein Interaction Networks
Source: PLoS One. 2013 Feb 19;8(2):e56833. doi: 10.1371/journal.pone.0056833 (PMC3576370; doi:10.1371/journal.pone.0056833)
Supplement: Table S2 — Accuracy, precision, recall, and correlation coefficient (CC) of classification for the protein-binding versus non-protein-binding dataset are presented for standard machine learning methods. For each machine learning approach, values of k ranged from 1 to 2. The performances of the results were estimated using cross-validation. The highest performing value(s) for each performance measure is highlighted in bold. (DOCX) [file pone.0056833.s004.docx]

**Table S2.** Dataset 1 results on standard machine learning methods. Accuracy, precision, recall, and correlation coefficient (CC) of classification for the protein-binding versus non-protein-binding dataset are presented for standard machine learning methods. For each machine learning approach, values of k ranged from 1 to 2. The performances of the results were estimated using cross-validation. The highest performing value(s) for each performance measure is highlighted in bold.

| Approach | k | Accuracy | Precision | Recall | CC |
| --- | --- | --- | --- | --- | --- |
| Decision Tree | 1 | 81.6 | .72 | .68 | .57 |
|  | 2 | 74.4 | .60 | .60 | .41 |
| SVM | 1 | 85.2 | .83 | .67 | .64 |
|  | 2 | 87.2 | .82 | .76 | .70 |
| ANN | 1 | 85.4 | .81 | .70 | .65 |
|  | 2 | 86.9 | .83 | .73 | .71 |
| Naïve Bayes | 1 | 81.8 | .72 | .70 | .58 |
|  | 2 | 82.1 | .70 | .76 | .60 |
| **HybSVM** | **N/A** | **94.2** | **.92** | **.89** | **.87** |
