# Supplementary material for: Predicting the Binding Patterns of Hub Proteins: A Study Using Yeast Protein Interaction Networks
Source: PLoS One. 2013 Feb 19;8(2):e56833. doi: 10.1371/journal.pone.0056833 (PMC3576370; doi:10.1371/journal.pone.0056833)
Supplement: Table S3 — Accuracy, precision, recall, and correlation coefficient (CC) of classification for the multi-interface versus singlish-interface dataset are presented for internal machine learning methods. For each machine learning approach, values of k ranged from 1 to 4. The performances of the results were estimated using cross-validation. The highest performing value(s) for each performance measure is highlighted in bold. (DOCX) [file pone.0056833.s005.docx]

**Table S3.** Dataset 3 results on our internal machine learning methods. Accuracy, precision, recall, and correlation coefficient (CC) of classification for the multi-interface versus singlish-interface dataset are presented for internal machine learning methods. For each machine learning approach, values of k ranged from 1 to 4. The performances of the results were estimated using cross-validation. The highest performing value(s) for each performance measure is highlighted in bold.

| Approach | k | Accuracy | Precision | Recall | CC |
| --- | --- | --- | --- | --- | --- |
| NB k-gram | 1 | 72.9 | .37 | .39 | .20 |
|  | 2 | 78.7 | .48 | .53 | .36 |
|  | 3 | 82.5 | .25 | **.90** | .42 |
|  | 4 | 83.8 | .42 | .75 | .47 |
| NB(k) | 2 | 81.9 | .51 | .62 | .44 |
|  | 3 | 83.2 | .31 | .84 | .44 |
|  | 4 | 69.6 | .74 | .40 | .35 |
| Domain-based | N/A | 76.4 | .00 | .00 | -.01 |
| Homology-based | N/A | 66.4 | .74 | .34 | .32 |
| **HybSVM** | **N/A** | **89.0** | **.75** | .77 | **.69** |
