# Supplementary material for: Predicting the Binding Patterns of Hub Proteins: A Study Using Yeast Protein Interaction Networks
Source: PLoS One. 2013 Feb 19;8(2):e56833. doi: 10.1371/journal.pone.0056833 (PMC3576370; doi:10.1371/journal.pone.0056833)
Supplement: Table S4 — Accuracy, precision, recall, and correlation coefficient (CC) of classification for the multi-interface versus singlish-interface dataset are presented for standard machine learning methods. For each machine learning approach, values of k ranged from 1 to 3. The performances of the results were estimated using cross-validation. The highest performing value(s) for each performance measure is highlighted in bold. (DOCX) [file pone.0056833.s006.docx]

**Table S4.** Dataset 3 results on standard machine learning methods. Accuracy, precision, recall, and correlation coefficient (CC) of classification for the multi-interface versus singlish-interface dataset are presented for standard machine learning methods. For each machine learning approach, values of k ranged from 1 to 3. The performances of the results were estimated using cross-validation. The highest performing value(s) for each performance measure is highlighted in bold.

| Approach | k | Accuracy | Precision | Recall | CC |
| --- | --- | --- | --- | --- | --- |
| Decision Tree | 1 | 71.6 | .30 | .24 | .07 |
|  | 2 | 67.7 | .32 | .37 | .13 |
|  | 3 | 71.0 | .38 | .43 | .21 |
| SVM | 1 | 77.4 | .00 | .00 | .00 |
|  | 2 | 76.1 | .46 | .37 | .27 |
|  | 3 | 80.6 | **.86** | .17 | .23 |
| ANN | 1 | 76.7 | .00 | .00 | .00 |
|  | 2 | 78.0 | .56 | .09 | .08 |
|  | 3 | 76.7 | .38 | .05 | .03 |
| Naïve Bayes | 1 | 70.3 | .40 | .63 | .29 |
|  | 2 | 73.5 | .43 | .49 | .28 |
|  | 3 | 81.2 | .62 | .46 | .41 |
| **HybSVM** | **N/A** | **89.0** | .75 | **.77** | **.69** |
