# Supplementary material for: Predicting the Binding Patterns of Hub Proteins: A Study Using Yeast Protein Interaction Networks
Source: PLoS One. 2013 Feb 19;8(2):e56833. doi: 10.1371/journal.pone.0056833 (PMC3576370; doi:10.1371/journal.pone.0056833)
Supplement: Table S5 — Accuracy, precision, recall, and correlation coefficient (CC) of classification for the date versus party dataset are presented for internal machine learning methods. For each machine learning approach, values of k ranged from 1 to 4. The performances of the results were estimated using cross-validation. The highest performing value(s) for each performance measure is highlighted in bold. (DOCX) [file pone.0056833.s007.docx]

**Table S5.** Dataset 4 results on our internal machine learning methods. Accuracy, precision, recall, and correlation coefficient (CC) of classification for the date versus party dataset are presented for internal machine learning methods. For each machine learning approach, values of k ranged from 1 to 4. The performances of the results were estimated using cross-validation. The highest performing value(s) for each performance measure is highlighted in bold. Naïve Bayes is abbreviated as NB.

| Approach | k | Accuracy | Precision | Recall | CC |
| --- | --- | --- | --- | --- | --- |
| NB k-gram | 1 | 64.1 | .48 | .64 | .27 |
|  | 2 | 66.1 | .49 | **.68** | .31 |
|  | 3 | 67.1 | .54 | .67 | .33 |
|  | 4 | 60.6 | .53 | .57 | .20 |
| NB(k) | 2 | 64.6 | .45 | .67 | .28 |
|  | 3 | 65.1 | .53 | .64 | .29 |
|  | 4 | 57.5 | .58 | .53 | .15 |
| Domain-based | N/A | 59.1 | .62 | .30 | .14 |
| Homology-based | N/A | 29.8 | .22 | .22 | -.43 |
| **HybSVM** | **N/A** | **69.2** | **.71** | .56 | **.37** |
