# Supplementary material for: Predicting the Binding Patterns of Hub Proteins: A Study Using Yeast Protein Interaction Networks
Source: PLoS One. 2013 Feb 19;8(2):e56833. doi: 10.1371/journal.pone.0056833 (PMC3576370; doi:10.1371/journal.pone.0056833)
Supplement: Table S6 — Accuracy, precision, recall, and correlation coefficient (CC) of classification for the date versus party dataset are presented for standard machine learning methods. For each machine learning approach, values of k ranged from 1 to 3. The performances of the results were estimated using cross-validation. The highest performing value(s) for each performance measure is highlighted in bold. (DOCX) [file pone.0056833.s008.docx]

**Table S6.** Dataset 4 results on standard machine learning methods. Accuracy, precision, recall, and correlation coefficient (CC) of classification for the date versus party dataset are presented for standard machine learning methods. For each machine learning approach, values of k ranged from 1 to 3. The performances of the results were estimated using cross-validation. The highest performing value(s) for each performance measure is highlighted in bold.

| Approach | k | Accuracy | Precision | Recall | CC |
| --- | --- | --- | --- | --- | --- |
| Decision Tree | 1 | 53.5 | .50 | **.62** | .08 |
|  | 2 | 51.0 | .46 | .43 | .01 |
|  | 3 | 52.0 | .48 | .48 | .03 |
| SVM | 1 | 62.1 | .61 | .50 | .23 |
|  | 2 | 58.1 | .55 | .52 | .15 |
|  | 3 | 62.1 | .59 | .59 | .24 |
| ANN | 1 | 64.6 | .69 | .42 | .27 |
|  | 2 | 66.2 | .70 | .46 | .30 |
|  | 3 | 65.2 | .67 | .47 | .28 |
| Naïve Bayes | 1 | 65.2 | .66 | .51 | .29 |
|  | 2 | 64.1 | .65 | .48 | .26 |
|  | 3 | 62.6 | .61 | .52 | .24 |
| **HybSVM** | **N/A** | **69.2** | **.71** | .56 | **.37** |
