# Supplementary material for: Predicting the Binding Patterns of Hub Proteins: A Study Using Yeast Protein Interaction Networks
Source: PLoS One. 2013 Feb 19;8(2):e56833. doi: 10.1371/journal.pone.0056833 (PMC3576370; doi:10.1371/journal.pone.0056833)
Supplement: Table S7 — The formula for binary classification for each of our five performance measures is provided. TP , TN , FP , FN are the true positives, true negatives, false positives, and false negative predictions. (DOCX) [file pone.0056833.s009.docx]

**Table S7.**Performance measures. The formula for binary classification for each of our five performance measures is provided. *TP, TN, FP, FN* are the true positives, true negatives, false positives, and false negative predictions.

| **Performance Measure** | **Formula for Binary Classification** |
| --- | --- |
| Accuracy | $\frac{TP+TN}{TP+FP+TN+FN}$ |
| Precision | $\frac{TP}{TP+FP}$ |
| Recall | $\frac{TP}{TP+FN}$ |
| Correlation Coefficient | $\frac{\left(TP\times TN\right)-\left(FP\times FN\right)}{\sqrt{(TP+FN)(TP+FP)(TN+FP)(TN+FN)}}$ |
| F-measure | $2\left(\frac{precision\times recall}{precision+recall}\right)$ |
